# Risk factors for revision after shoulder arthroplasty: 1,825 shoulder arthroplasties from the Norwegian Arthroplasty Register

**DOI:** 10.1080/17453670902805098

**Published:** 2009-02-01

**Authors:** Bjørg-Tilde S Fevang, Stein A Lie, Leif I Havelin, Arne Skredderstuen, Ove Furnes

**Affiliations:** ^1^Department of Rheumatology and The Norwegian Arthroplasty Register, Department of Orthopedic Surgery, Haukeland University HospitalBergenNorway; ^2^The Norwegian Arthroplasty Register, Department of Orthopedic Surgery, Haukeland University HospitalBergenNorway; ^3^Section of Orthopedic Surgery, Department of Surgical Sciences, University of Bergen and Department of Health, University Research BergenBergenNorway; ^4^Department of Health, University Research BergenBergenNorway

## Abstract

**Background and purpose** Previous studies on shoulder arthroplasty have usually described small patient populations, and few articles have addressed the survival of shoulder implants. We describe the results of shoulder replacement in the Norwegian population (of 4.7 million) during a 12-year period. Trends in the use of shoulder arthroplasty during the study period were also investigated.

**Patients and methods** 1,531 hemiprostheses (HPs), 69 total shoulder replacements (Neer type TSR), and 225 reversed total shoulder replacement (reversed TSR) operations were reported to the Norwegian Arthroplasty Register between 1994 and 2005. Kaplan-Meier failure curves were drawn up for particular subgroups of patients, and revision rates were calculated using Cox regression analysis.

**Results** The 5- and 10-year failure rates of hemiprostheses were 6% (95% CI: 5–7) and 8% (95% CI: 6–10), and for reversed total shoulder replacements they were 10% (95% CI: 5–15) and 22% (95% CI: 10–33), respectively. For hemiprostheses, the risk of revision for patients who were 70 years or older was half that of those who were younger (RR = 0.47, CI: 0.28–0.77), while the risk of revision was highest for patients with sequelae after fracture compared to those with acute fractures (RR = 3.3, CI: 1.5–7.2). No differences in prosthesis survival were found between the different hemiprosthesis brands. The main reasons for revision of hemiprostheses were pain and luxation. For reversed total prostheses, the risk of revision was less for women than for men (RR = 0.26, CI: 0.11–0.63), and the main cause of revision was aseptic loosening of the glenoid component. During the study period, the incidence of shoulder arthroplasty increased for all diagnostic groups except inflammatory arthritis, for which a decrease was seen.

**Interpretation** We found good results in terms of 5-year prosthesis failure rate, with the use of hemiarthroplasty for patients with inflammatory arthritis, osteoarthritis, and acute fractures. Reversed total shoulder replacement was associated with a rather poor prognosis.

## Introduction

Shoulder replacement surgery was introduced as a treatment method for 3- or 4-part proximal humerus fractures by Neer in the 1950s (Neer et al. 1953). During the 1970s, the use of shoulder arthroplasty was extended to include patients with osteoarthritis (Neer 1974), and this treatment method was eventually used with success in other diagnostic groups such as inflammatory arthritis.

Loosening of the glenoid component has been shown to be a major problem in total shoulder arthroplasty (Cofield and Edgerton 1990, Torchia et al. 1997, Bohsali et al. 2006). However, pain due to glenoid arthritis has been described after hemiarthroplasty (Parsons et al. 2004, Haines et al. 2006). Previous studies on revision after shoulder arthroplasty have usually included small samples, and they have rarely compared survival between groups. In a study of total shoulder arthroplasty, the survival rates were 95% at 5 years and 85% at 10 years (Martin et al. 2005). Haines et al. (2006) found different survival rates according to the primary diagnosis, the 10-year survival being 86% for patients with osteoarthritis and 33% for posttraumatic arthritis. In proximal humerus fractures, survival of the prosthesis has been reported to be 83–94% at 10 years (Robinson et al. 2003, Kwon and Zuckerman 2005), and a study of patients with rheumatoid arthritis reported prosthesis survival at 8 years to be 92% (Trail and Nuttall 2002).

We evaluated prosthesis failure after shoulder replacement in a large national series of patients, and we investigated the effect of prosthesis type and shoulder disease on the outcome. Furthermore, we assessed trends in the use of shoulder arthroplasty over a 12-year period.

## Patients and methods

The Norwegian Arthroplasty Register (NAR) was established in 1987, initially as a hip prosthesis register. In January 1994, it was extended to include all arepsicial joints (Havelin 1999). Individual reports of joint replacements were received from all the hospitals in Norway that were performing arthroplasty surgery. Data concerning identity of the patient, diagnosis, date of surgery, type of prosthesis, whether cement was used, whether the operation was primary or a revision, cause of revision, and procedure at revision were taken from the form filled in by the operating surgeon (Furnes et al. 2002). The completeness of registration was evaluated in a recent study and 89% of all primary shoulder arthroplasty operations were reported to the NAR (Espehaug et al. 2006). In the present study involving 1,825 shoulder arthroplasties, the main outcome variable was whether or not a revision was performed after primary surgery. No information on function or patient satisfaction was registered.

From 1994 through 2005, 1,531 hemiprosthesis procedures and 294 total shoulder replacement procedures were reported to the NAR. A reversed total prosthesis was used in 225 cases and a Neer-type total prosthesis was used in 69 cases. Mean age of the patients operated with a reversed prosthesis was 69 years, which was practically the same as in the total material (Table 1). There were 1,428 women and 397 men, and the mean age at surgery was significantly higher in women (70 years for women as opposed to 64 years for men) (p < 0.001).

**Table 1.** General characteristics and diseases leading to shoulder replacement

Diagnosis **^a^**	Total no. of patients	Hemiprosthesis	Reversed TSR	Neer-type TSR
Total	1,820	1,526	225	69
Age (mean, SD)	69 (12)	69 (13)	69 (11)	68 (11)
Sex (% women)	78%	78%	83%	69%
Diagnosis				
Osteoarthritis	427	338	50	39
Rheumatoid arthritis	569	439	118	12
Acute fracture	426	422	4	–
Fracture sequelae	350	303	34	13
Ankylosing spondylitis	16	14	1	1
Psoriatic arthritis	18	15	3	–
Rotator cuff /ligament damage	23	10	13	–
Other **^b^**	64	48	11	5

**^a^**More than one diagnosis was allowed.

**^b^**Avascular necrosis, sequelae after infection, hemophilia, cancer, sequelae after luxation, SLE, chondrocalcinosis, sequelae after frozen shoulder, synovial chondromatosis, and unknown.

During the study period, 13 different brands of shoulder prostheses were used (Table 2). The most commonly used type, the “Neer-type”, consisted of a stem and a caput and, for total shoulder replacements, a glenoid component. They were inserted with or without cement. The Delta I prosthesis differs from the others in having a hydroxyapatite-coated surface.

**Table 2.** Prosthesis brands

Prosthesis brand	HP	Neertype TSR	Reversed TSR	Hospitals using the HP	Hospitals with > 10 HPs **^a^**	Hospitals using the TSR	Hospitals with > 10 TSRs **^a^**
Bio-Modular (Biomet)	604	48		26	14 (7)	9	2
Global (DePuy)	261	1		21	8 (3)	1	0
Delta III (DePuy)			225			17	6 (3)
Copeland (Biomet)	121	4		15	3 (2)	3	0
Nottingham (Biomet)	109	13		4	2 (2)	1	1
Global Advantage (DePuy)	105	1		15	1 (1)	1	0
Global Fx(DePuy)	119			17	0		
Delta I (Medinov)	58			7	2 (1)		
Scan Shoulder (Mitab)	56			7	2		
Neer II (3M Healthcare)	40			6	1		
Modular (3M Healthcare)	33			1	1 (1)		
Monospherical (Howmedica)	13	1		3	0	1	0
Bigliani Flatow (Zimmer)	6			2	0		
	1,525 **^b^**	68 **^b^**	225				

HP: hemiprosthesis; TSR: total shoulder replacement.

**^a^** Number in parentheses: hospitals with > 30 procedures

**^b^** For one TSR and one HP, the brand of prosthesis was not given.

The Delta III total shoulder replacement (DePuy International Ltd. Leeds, England) represents a separate entity using the reversed design with a glenoid head and humeral cup. The Copeland prostheses (Biomet Merck Limited, South Wales, UK) as well as 42 of the Scan prostheses were resurfacing prostheses.

132 patients were given shoulder replacements bilaterally during the study period. In these patients, each replacement procedure was considered a separate case. A revision (or failure) was defined as the removal or exchange of part of or the whole implant. The follow-up time was the time from primary shoulder replacement until revision, or until the end of the study or death. The date of death for the patients was obtained from Statistics Norway (www.ssb.no/english/). Median follow-up time was 4.0 years (range: 2 days to 12 years).

Major causes of surgery were rheumatoid arthritis (RA), osteoarthritis (OA), acute fracture, and fracture sequelae (Table 1). Only 4 patients with acute fractures had a TSR, while TSR was used more commonly for the other major diagnostic groups.

**Figure 1.**
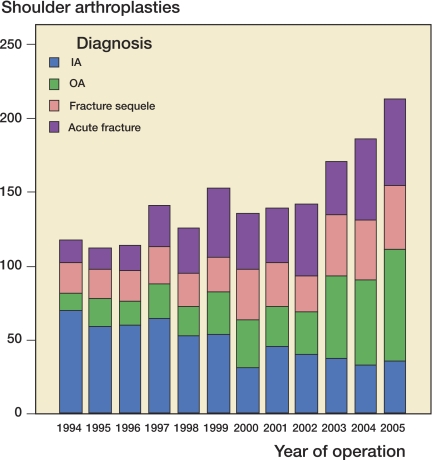
Shoulder arthroplasties in 4 major diagnostic groups from 1994 through 2005. IA: inflammatory arthritis; OA: osteoarthritis.

In some analyses (Table 4, Figures 1 and 3), rheumatoid arthritis, psoriatic arthritis, and ankylosing spondylitis were grouped together and called inflammatory arthritis (IA). Several causes of revision could be given for the same patient (Table 5). For the purpose of this study, however, pain was only registered as the cause of revision in cases where this was the only recorded cause.

### Statistics

The Student t-test was used to compare continuous variables. The significance level was set at 5% and all p-values were two-tailed. Median follow-up (observation) time was calculated using the reverse Kaplan-Meier method. Kaplan-Meier survival tables were used to find 5- and 10-year failure rates, calculated as 1 minus the survival rate. Failure curves based on the Kaplan-Meier method with revision for any reason as endpoint were given for several subgroups of patients. The failure curves were discontinued when the number of patients at risk was less than 20. In failure curves for different brands of hemiprosthesis, only brands that had been used in at least 30 shoulders were included in the analyses (Figure 3a). The curve for Global Fx was not shown because the follow-up time was short (median 1.3 years).

Cox multiple regression analysis was used to calculate relative risk (RR) of revision according to shoulder disease, age, sex, and year of primary operation. Separate analyses were done for hemiprostheses and reversed total shoulder replacements (Table 5). All relative risks were adjusted for the other variables in the analysis. A Cox regression analysis was also done to compare resurfacing hemiprostheses to other hemiprostheses. The analysis is not shown, but the p-value given in the text was obtained from this analysis.

For revision due to specific causes (such as aseptic loosening or infection), the number of events was small with occasional zeros (Table 6), which is why models for exact Cox regression were set up. The statistical program LogXact (Cytel Inc., Cambridge, MA) was used according to Samuelsen (2003). The models were adjusted for shoulder disease, cement, sex, and age.

Poisson regression analysis was used to analyze trends in the incidence of shoulder arthroplasties for all shoulder arthroplasties and for HP and TSR separately (Table 3), and for the 4 major diagnostic groups (Figure 1). These analyses were performed based on annual population rates for the Norwegian population obtained from Statistics Norway. The p-values given in Table 3 and in the legend to Figure 1 were derived from these Poisson analyses. All analyses, except for the exact regression analyses (LogXact), were performed using SPSS software version 13.0.

**Table 3.** Primary shoulder arthroplasties per annum. Overall incidence (per 100,000 inhabitants), total annual number, and annual number according to prosthesis type

Year	Overall incidence	All primary operations	Hemiprostheses	Total prostheses
1994	2,80	121	93	28
1995	2,67	116	87	29
1996	2,77	121	94	27
1997	3,37	148	133	15
1998	2,90	128	118	10
1999	3,55	158	135	23
2000	3,13	140	114	26
2001	3,24	146	130	16
2002	3,34	151	122	29
2003	3,98	181	153	28
2004	4,33	198	168	30
2005	4,71	217	184	33
Total		1820	1526	294
p-value **^a^**	< 0.001	< 0.001	< 0.00	0.31

**^a^** p-value calculated using Poisson regression analysis based on annual population rates for the Norwegian population.

## Results

### Time trends

An increase in the overall incidence of shoulder arthroplasties took place during the 12-year study period, and this trend was seen for HP but not for TSR (p < 0.001 and p = 0.3) (Table 3). The increasing trend was seen in all the major diagnostic groups except inflammatory arthritis, for which the trend was opposite (p < 0.001) (Figure 1).

### Operation volume

The shoulder replacements were performed at 54 hospitals. On average, 4 procedures were done per hospital per year, and only 2 hospitals had 10 or more procedures annually. The mean number of HP operations performed per hospital during the study period was 28 (1–176) while for reversed TSR the mean was 13 (1–74) procedures per hospital that performed TSR (p = 0.01). 7 HP brands and 1 TSR brand had a relatively high volume in at least one hospital (> 30 cases during the study period) (Table 1).

### Revision procedures

The procedures performed at revision after TSR were exchange of parts of or the whole prosthesis in 22 patients, and removal of prosthetic parts without replacement in 14 cases. In 2 cases, the procedure performed during revision was not reported. In patients with HP, removal of prosthesis or prosthesis components was performed in 11 cases, while in 61 patients the whole prosthesis or parts were exchanged. The procedure was not reported in 3 cases with HP.

### Revision after hemiarthroplasty

**Figure 2.**
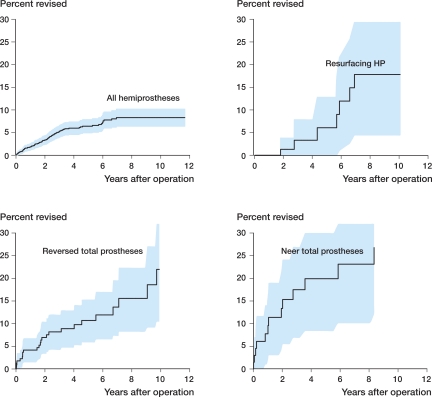
Kaplan-Meier failure curve (with 95% CI shown in color) for all hemiprostheses, resurfacing hemiprostheses, reversed total shoulder replacement, and Neer-type total shoulder replacement.

**Figure 3.**
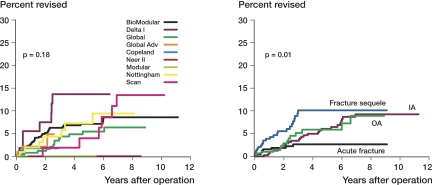
A. Kaplan-Meier failure curves for different hemiprosthesis brands. Groups with less than 20 patients were excluded. B. Kaplan-Meier failure curves according to shoulder disease.

The cumulative 5- and 10-year failure rates for HP were 6% and 8% (Figure 2). When analyzing resurfacing hemiprostheses separately, there was no statistically significant difference between these prostheses and other HPs (p = 0.9) (Figure 2). No statistically significant difference in failure rate was found between the different brands of hemiprosthesis (Figure 3a). Shoulder disease did, however, influence the failure rate, and for hemiprostheses the failure rate was lowest for patients with an acute fracture and highest after fracture sequelae (p = 0.01) (Figure 3b). Cox regression analysis showed that the relative risk of revision was 3.3 (CI: 1.5–7.2) for patients with sequelae after fractures compared to those with acute fractures (Table 4a).

**Table:** Table 4a. Cox regression analysis for revision after hemiarthroplasty, according to potential explanatory factors. 5- and 10-year failure rates estimated using the Kaplan-Meier method

	No.	No. of revisions	F_5_ (%)	95% CI	F_10_ (%)	95% CI	RR	95% CI	p-value
All	1,470 **^a^**	74	6	5–7	8	6–10			
Age									
0–69 years	664	49	9	7–12	12	9–15	1		
≥ 70 years	806	25	4	3–5	4	3–5	0.47	0.28–0.77	0.003
Sex									
Men	312	14	6	3–10	8	4–12	1		
Women	1158	60	6	5–8	8	6–10	1.5	0.82–2.7	0.2
Year of surgery							1.0	0.92–1.1	1
Diagnosis									
Acute fracture	422	9	1	0–2	3	1–4	1		
IA **^b^**	440	28	6	3–8	9	6–12	1.8	0.80–3.9	0.1
OA **^b^**	311	14	6	3–9	9	4–14	2.2	0.95–5.1	0.07
Fracture									
sequelae	297	23	10	6–14	10	6–14	3.3	1.5–7.2	0.002

**^a^**61 ca ses were not included due to missing values or due to the fact that the patient had a shoulder disease other than the 4 included in this analysis.

**^b^**OA: osteoarthritis; IA: inflammatory arthritis including RA, psoriatic arthritis, and ankylosing spondylitis.

F5 and F10: unadjusted Kaplan-Meier 5-year and 10-year failure rates.

RR: relative risk of revision derived from the Cox model.

The median follow-up time was longer for patients with IA (6.1 years) than for those with OA (2.9 years), acute fracture (3.3 years), and sequelae after fracture (3.5 years) (p < 0.001).

Furthermore, patients who were 70 years old or more had half the risk of revision compared to those who were younger (RR = 0.47, CI: 0.28–0.77) (Table 4a), while the risk of revision was independent of gender and year of operation. Cementation of the humeral component had no influence on revision rate (RR = 1.1, CI = 0.67–1.8) (not presented in the table).

### Revision after total shoulder arthroplasty

The 5- and 10-year failure rates for the reversed TSR were 10% and 22% (Figure 2). The failure curve for Neer-type TSR shows 5- and 10-year revision rates of 20% and 27% (Figure 2). The Cox regression analysis did not reveal any difference in revision rates between the 3 major diagnostic groups for reversed TSR (Table 4b). Women had a lower risk of revision than men (RR = 0.26, CI: 0.11–0.63) (Table 4b).

**Table:** Table 4b. Cox regression survival analysis for revision after reversed TSR, according to potential explanatory factors. 5- and 10-year failure rates estimated using the Kaplan-Meier method

	No.	No. of	F_5_ (%) revisions	95% CI	F_10_ (%)	95% CI	RR	95% CI	p-value
All		201 **^a^**	22	10	85–95	22	67–90		
Age									
0–69 years	93	13	11	4–18	26	11–41	1		
≥ 70 years	108	9	8	3–14	12	3–20	0.82	0.32–2.12	0.7
Sex									
Men	32	9	30	11–50	51	23–80	1		
Women	169	13	7	2–11	15	4–26	0.26	0.11–0.63	0.003
Year of surgery						1.09	0.93–1.3	0.3	
Diagnosis									
IA **^b^**	120	14	9	3–14	20	8–32	1		
OA **^b^**	47	6	14	1–28	36	0–73	1.49	0.52–4.3	0.5
Fracture sequelae	34	2	10	0–23	10	0–23	0.64	0.14–2.9	0.6

**^a^** 24 cases were not included due to missing values or due to the fact that the patient had a shoulder disease other than the 3 included in this analysis.

**^b^** OA: osteoarthritis; IA: inflammatory arthritis including RA, psoriatic arthritis, and ankylosing spondylitis.

F5 and F10: unadjusted Kaplan-Meier 5-year and 10-year failure rates.

RR: relative risk of revision derived from the Cox model.

### Causes of revision

The main cause of revision after TSR was aseptic loosening of the glenoid component (Table 5). For hemiprostheses, the major cause of revision was pain, seen in 15 of 439 cases with RA but in none of the 422 cases with acute fractures. Altogether, this was given as the only cause of revision in 32 cases. Other major causes of revision are listed in Table 5.

**Table 5.** Causes of revision, according to type of prosthesis

Cause **^a^**	All (n = 1,820)	Hemiprosthesis (n = 1,526)	TSR reversed (n = 225)
Aseptic loosening of glenoid component	17	–	13
humeral component	11	6	5
Luxation	21	11	4
Instability	9	6	2
Deep infection	11	5	5
Fracture	3	2	1
Pain	34	32	2
Failure of function	3	3	–
Other **^b^**	26	20	4

**^a^** More than one cause may have been given, but pain was included only when it was given as the sole cause of revision.

**^b^** Including incorrect axis, broken prosthesis component, component too small or too large, subluxation, and other not specified.

For hemiprostheses, revision due to pain was less frequent in patients with acute fractures (RR = 0.12, p = 0.02) and in patients > 70 years of age (RR = 0.25, p = 0.005) (Table 6a). In HP patients with sequelae after fractures, revision due to instability and luxation was more common (RR = 11, p = 0.04 and RR = 8.8, p = 0.04, respectively) (Table 6a).

**Table:** Table 6a. Risk of revision due to specific causes, for hemiprostheses (HPs), according to shoulder disease and fixation. The results were obtained using an exact regression model

	Aseptic loosening			Pain **^d^**			Deep infection			Instability			Luxation of prosthesis		
	n **^a^**	RR **^b^** (95% CI)	p	n **^a^**	RR **^b^** (95% CI)	p	n **^a^**	RR **^b^** (95% CI)	p	n **^a^**	RR **^b^** (95% CI)	p	n **^a^**	RR **^b^** (95% CI)	p
Shoulder disease															
IA **^c^**(439)	4	1		15	1	1	1			0	1		0	1	
OA **^c^** (311)	2	1.1	1.0	5	1.0	1.0	2	2.5	0.87	1	4.2	0.38	2	2.8	0.4
		(0.09–9.0)			(0.28–3.1)			(0.12–156)			(0.11–∞)			(0.22–∞)	
Sequelae of fracture (296)	1	0.53	0.99	8	1.5	0.52	1	1.4	1.0	3	11.4	0.04	5	8.8	0.04
		(0.01–5.7)			(0.53–3.8)			(0.02–119)			(1.1–∞)			(1.1–∞)	
Acute fracture (421)	0	0.28	0.27	0	0.12	0.02	0	1.1	1.0	1	1.8	0.72	2	1.7	0.7
		(0.0–2.5)			(0.0–0.80)			(0.0–41)			(0.05–∞)			(0.13–∞)	
Fixation															
Cemented (944)	5	1		10	1		2	1		3	1			6	1
Uncemented (523)	2	0.28	0.24	18	1.4	0.56	2	1.2	1.0	2	0.91	1.0	3	1.4	0.9
		(0.03–1.8)			(0.59–3.4)			(0.08–17)			(0.07–9.9)			(0.21–7.6)	

In the analyses, some cases were excluded due to missing values (i.e. patients with diagnoses other than the major ones).

**^a^**Number of revisions due to each cause.

**^b^** Adjusted for age and sex. Revision due to pain was significantly less frequent in patients who were 70 years or older compared to those who were younger (RR = 0.25, p = 0.005).

**^c^** OA: osteoarthritis; IA: inflammatory arthritis including RA, psoriatic arthritis, and ankylosing spondylitis.

**^d^** Pain was registered only when it was given as the only cause of revision.

For reversed TSR, women had a smaller risk of revision due to deep infections than men (RR = 0.006, p = 0.01) (Table 6b). Except for this, no statistically significant differences in revision due to the causes given in Table 6, were found between patients with different shoulder diseases and fixation methods.

**Table:** Table 6b. Risk of revision due to specific causes, after reversed TSR, according to shoulder disease and fixation. The results were obtained using an exact regression model

	Aseptic loosening			Pain **^d^**			Deep infection			Instability			Luxation of prosthesis		
	n **^a^**	RR **^b^** (95% CI)	p	n **^a^**	RR **^b^** (95% CI)	p	n **^a^**	RR **^b^** (95% CI)	p	n **^a^**	RR **^b^** (95% CI)	p	n **^a^**	RR **^b^** (95% CI)	p
Shoulder disease															
IA **^d^**(120)	9	1		1	1		2	1		1	1		2	1	
OA **^d^** (47)	3	1.6	0.8	1	2.2	1	0	2.0	1	1	1.3	1.0	2	2.1	0.9
		(023–8.4)			(0.03–164)			(0.0–26)			(0.02–106)			(0.13–38)	
Sequelae of fracture (296)	1	0.62	1	0	4.3	1	1	1.8	1	0	6.5	1.0	0	1.4	1
		(0.01–5.7)			(0.0–166)			(0.03–35)			(0.0–252)			(0.00–18)	
Fixation															
Cemented (61)	1	1		1	1		1	1		0	1		2	1	
Uncemented (264)	13	1.5	1	1	0.08	0.4	4	0.15	1	2	0.68	1.0	2	0.31	0.5
		(0.19–68)			(0.0007–9.4)			(0.0004–∞)			(0.05–∞)			(0.02–4.6)	

In the analyses, some cases were excluded due to missing values (i.e. patients with diagnoses other than the 3 major ones).

**^a^** Number of revisions due to each cause.

**^b^** Adjusted for age and sex. Revision due to deep infection was significantly less frequent in women than in men (RR = 0.06, p = 0.01).

**^c^** Loosening of humeral or glenoid component, or both.

**^d^** OA: osteoarthritis; IA: inflammatory arthritis including RA, psoriatic arthritis, and ankylosing spondylitis.

**^e^** Pain was registered only when it was given as the only cause of revision.

## Discussion

Our patient series is among the largest in studies on hemiprostheses of the shoulder. The major finding was that the survival after insertion of a hemiprosthesis was satisfactory, with 6% and 8% revision within 5 and 10 years, respectively. For the reversed TSR, however, the results were more disappointing with more than 20% revision at 10 years.

The age and sex distribution was similar to that in some other large series (Jain et al. 2004, Sharma and Dreghorn 2006). In our study, there were 84% hemiprostheses, which is similar to the findings from the Scottish and Swedish registries (88% and 87%, respectively) (Rahme et al. 2001, Sharma and Dreghorn 2006). Ravenscroft and Calvert (2004) reported twice as many HPs as TSRs in the UK, but there were more TSRs in a large American study (Jain et al. 2004).

### Time trends

There was an increase in the overall incidence of shoulder arthroplasty during the 12-year period. A similar trend was recently described in a review by Bohsali et al. (2006). In the Norwegian population, the increased incidence only applied to hemiprostheses while no significant change in the incidence of total shoulder replacement (TSR) was detected. Jain et al., investigating time trends for TSR in the United States, found a minor increase in the use of TSR during the period 1990–2000 (Jain et al. 2006).

We found an increase in the use of shoulder replacement for patients with osteoarthritis, fracture sequelae, and acute fractures, but the opposite trend was seen for patients with IA. This corresponds well with the trend in the US population reported by Jain et al. (2006), showing an increase in the use of TSR due to osteoarthritis and a minor decline for rheumatoid arthritis. The findings correspond well with the general trend towards reduced need for surgical procedures in patients with rheumatic diseases (Fevang et al. 2007).

### Operation volume

In a recent study of knee replacements, the revision rate was found to be lower at hospitals having a high volume of operations (Furnes et al. 2007). Jain et al. (2004) found better outcome in terms of in-hospital mortality and complications, length of stay, and disposition of patients on discharge, after shoulder arthroplasty performed in high-volume hospitals. In our study, none of the hospitals had a high volume of shoulder surgery, and the procedures at the “high-volume hospitals” may have been performed by several different surgeons. Still, the average number of procedures was lower for the reversed TSRs and this may have contributed to the inferior results.

### Revision after hemiarthroplasty

The overall 5-year failure rate for hemiprostheses (6%) in our study is acceptable; it is similar to the 4% overall 5-year failure rate for knee prostheses (Havelin et al. 2000) and 4% for hip prostheses (in patients with osteoarthritis) (Furnes et al. 2001). However, the threshold for revising a hip prosthesis may be different from that for shoulder prostheses. We have no information concerning the number of patients suffering from pain without being revised. Consequently, comparison of failure rates for different joints is difficult. Use of revision as outcome factor does, however, allow comparison of results for subgroups of patients with arthroplasties of the same joint.

The disease leading to the hemiarthroplasty influenced the outcome. Patients who were operated due to sequelae after a previous fracture had worse prognosis than patients with IA and acute fractures. Haines et al. (2006) also found the highest revision rate following shoulder arthroplasty in patients with posttraumatic arthritis. Sperling et al. (2007) reported a 5-year survival rate of 90% in 108 patients with RA who had a hemiarthroplasty, and Trail and Nuttall (2002) reported an 8-year survival of 92% in a study of 105 patients with rheumatoid arthritis who were treated with HP or TSR. Robinson et al. (2003) reported 94% prosthesis survival at 10 years in 163 patients treated with hemiarthroplasty due to proximal humeral fractures.

We found no statistically significant difference in failure rates for the different brands of hemiprostheses. Different brands would be best compared in randomized studies, but as far as we know, no such studies have been published. Register studies, however, have been proven useful in pointing out inferior brands of prosthesis or cement, due to the long observation time and large number of patients (Furnes et al. 1997, Espehaug et al. 2002). Thus, we may conclude that no particular brand of prosthesis had markedly inferior or superior results.

We found that the risk of revision was dependent on age. The failure rate was doubled in patients younger than 70 years, compared to those who were older. This has been shown previously for patients with hip and knee prostheses (Havelin et al. 1994, 2000), but not in shoulder arthroplasty.

### Revision after total shoulder arthroplasty

Previous studies on TSR have reported 10-year survival rates of between 93% and 97% (Torchia et al. 1997, Sperling et al. 2004, 2007, Deshmukh et al. 2005), which is much better than the 78% for reversed TSR in our study. Only Neer prostheses were used in these studies, as opposed to the reversed TSRs in our study. The survival results were, however, no better for the 69 Neer-type TSRs in our study. In a study of reverse total shoulder replacements, the 5-year survival was 91%, with replacement of the prosthesis as endpoint (Guery et al. 2006). These results are similar to ours, in which the 5-year survival for reversed total prostheses was 90%. The failure rate was higher for men than for women. A similar finding has been reported previously for total hip replacement (Havelin et al. 1994).

The indication for choosing one or other prosthesis is unknown in register studies, but as hemiarthroplasty was “the treatment of choice”, we cannot exclude the possibility that it was used in most “uncomplicated” cases—possibly contributing to the superior results with HP compared to TSR. Furthermore, the volume of TSR surgery at each hospital was lower than for HP. Surgeons’ experience in performing TSR is most probably less, and this may contribute to the inferior results for TSR. The use of reversed TSR is recommended for older patients, preferably 70 years or older (Matsen et al. 2007). In our study, the mean age at surgery was the same for reversed TSRs as for HPs (69 years). The use of reversed TSR in younger patients may, to some degree, have influenced the results adversely.

### Cause of revision

Glenoid loosening was the most common cause of revision in patients with reversed TSR (Table 5). This corresponds well with that reported in other studies of reversed TSR (Nwakama et al. 2000, Rittmeister and Kerschbaumer 2001, Sirveaux et al. 2004, Bohsali et al. 2006). In our study, the major cause of revision after insertion of hemiprostheses was pain. This was seen in 15 of 439 cases with RA, while none of the 422 cases with acute fracture had a revision due to pain. The reason for this is most likely that patients with IA often have some involvement of the glenoid, which may cause pain after insertion of a hemiprosthesis. The opposite is seen with acute proximal humeral fractures in which only the humerus is involved and the glenoid is unaffected.

### Strength and weaknesses of the study

One weakness of our study is that the use of different types and brands of prostheses might not have been randomly assigned. We have, however, adjusted for known possible confounding factors such as age, sex, year of surgery, and diagnosis, using regression analysis. Comparison of different subgroups should be made with caution, knowing that unknown factors may have influenced the results. The register has no data on function after shoulder arthroplasty. Thus, a follow-up study of postoperative function and pain in our patients is planned.

The use of registry data provides some advantages, in that we have almost complete data over a particular time period for a whole country, which represents a large study population. In addition, the results were obtained from all types of institutions—small and large—for all surgeons and for all diagnoses. Most other studies present the results of a single institution and often of a few specialized surgeons using one or a small number of prosthesis brands. The results of our study may be more easily generalized to the average hospital and surgeon. In register studies, revision is used as the outcome factor after joint replacement surgery. However, patients may suffer from pain but may still not have their implant replaced due to known difficulties during replacement surgery. Such situations may be different for different joints (i.e. hips versus shoulders), and also in patients with different diseases. Even so, a close relationship between rate of revision and the rate of radiographic failure has been shown previously (Fender et al. 1999), supporting the use of registry data (with revision as the outcome factor) for the surveillance of joint replacement surgery.

In summary, the failure rate after insertion of primary hemiprostheses was low. Patients with sequelae after fracture had a significantly higher risk of revision after hemiarthroplasty than patients with acute fracture or inflammatory arthritis. This might advocate more frequent use of HP for acute fractures, possibly reducing the need for arthroplasty due to sequelae after fractures. Reversed TSR was used in patients younger than 70 years of age, but this could not fully explain the relatively disappointing results with this type of prosthesis.
